# The parent–child relationship and adolescent alcohol use: a systematic review of longitudinal studies

**DOI:** 10.1186/1471-2458-12-886

**Published:** 2012-10-20

**Authors:** Leenke Visser, Andrea F de Winter, Sijmen A Reijneveld

**Affiliations:** 1 Department of Health Sciences, University Medical Center Groningen, University of Groningen, Antonius Deusinglaan 1, 9713, AV, Groningen, The Netherlands

**Keywords:** Alcohol use, Parent–child relationship, Longitudinal study, Child, Adolescent, Systematic review

## Abstract

**Background:**

Alcohol use among adolescents has become a major public health problem in the past decade and has large short- and long-term consequences on their health. The aim of this systematic review was to provide an overview of longitudinal cohort studies that have analyzed the association between the parent–child relationship (PCR) and change in alcohol use during adolescence.

**Methods:**

A search of the literature from 1985 to July 2011 was conducted in Medline, PsycINFO, and EMBASE in order to identify longitudinal, general population studies regarding the influence of the PCR on alcohol use during adolescence. The studies were screened, and the quality of the relevant studies was assessed. A best-evidence synthesis was used to summarize the results.

**Results:**

Twenty-eight relevant studies were identified. Five studies found that a negative PCR was associated with higher levels of alcohol use. Another seven papers only found this association for certain subgroups such as boys or girls, or a specific age group. The remaining sixteen studies did not find any association.

**Conclusions:**

We found weak evidence for a prospective association between the PCR and adolescent alcohol use. Further research to the association of the PCR with several types of alcohol use (e.g., initiation or abuse) and to the potential reversed causality of the PCR and alcohol use is required.

## Background

Alcohol use among adolescents has become a major public health problem over the past decade and can lead to a great many health risks and social problems. First, excessive alcohol use can reduce school performance [1]. Second, alcohol use increases the likelihood of being involved in fights, being injured, and injuring others [2]. Third, alcohol use is associated with relationship problems and risky sexual behavior [3].

There is extensive evidence supporting the association between the quality of the parent–child relationship (PCR) and child development [4]. PCR refers to parent or child appraisals of the quality of the relationship between them, characterized by parental behaviors which give evidence of a warm and supporting relationship (e.g., giving emotional affection or praising, active listening, encouraging or showing respect). This construct is sometimes measured as a negative PCR expressed in rejection, criticizing ideas frequently, having frequent arguments or withholding of affection [5,6]. Studies show that a negative PCR is related to externalizing problems such as aggressive and delinquent behavior [7,8]. Further, a negative PCR is related to an increased likelihood of internalizing problems such as depressive symptoms and anxiety [9,10] and even suicidal behavior [11].

There is also evidence that the PCR is related to adolescent alcohol drinking. Foxcroft and Lowe [12], Vakalahi [13], and Ryan et al. [14] have shown in their reviews that the PCR has a negative linear relationship with adolescent drinking.

The available reviews all have methodological shortcomings. A first one is that none of the previous reviews [12-14] did evaluate the methodological quality of the studies included. If summarizing the results of primary studies it is important to take into account their methodological quality because this may have an important impact on the results of systematic reviews [15-17] and on their implications and recommendations.

A second shortcoming of the available reviews was that they combined p-values to summarize findings [12,14] or only gave a descriptive summary [13]. A real synthesis of the best evidence helps to summarize the results taking into account the quality of the studies. This method draws conclusions based on the best available evidence or may conclude that conclusions cannot be drawn considering the currently available evidence [18].

A third shortcoming is that the reviews of Foxcroft and Lowe [12] and of Vakahali [13] included both cross-sectional and longitudinal studies, while longitudinal studies can provide more evidence of a causal association because the cause precedes the effect in time [19]. Although Ryan et al. [14] included only longitudinal studies they did not take into account whether or not previous alcohol use was accounted for in the analyses of the included studies. Controlling for the effects of previous alcohol use allows stronger statements to be made about the directionality of the association between the PCR and alcohol use.

For prevention strategies it is very relevant to study the influence of the PCR on changes in alcohol use in the general population. Since conclusive evidence on the relation between PCR and alcohol use is not available, the objective of the current review is to summarize and determine the strength of the evidence in terms of the effects that the PCR has on change in adolescent (defined from age 10 to 17) alcohol use. We included only longitudinal studies. To determine the influence that the PCR has on *change* in alcohol use, we reviewed only those studies in which previous alcohol use is accounted for in the analysis. We adhered to the PRISMA guidelines for reporting systematic reviews.

## Methods

### Study selection

An extensive search of the literature was conducted in order to identify longitudinal studies regarding the association between the PCR and adolescent alcohol use. The following databases were searched: Medline, PsycINFO, and EMBASE. The search was limited to studies which were published in the English language, during the period 1985-July 2011, and which focused on children or adolescents. The search strategy combined the following three sets of terms related to respectively alcohol use, PCR, and a longitudinal study design; (1) alcohol us* OR alcohol drink* OR alcohol dependen* OR alcohol abus* OR alcohol consum* OR binge drink* OR heavy drink* (textwords), OR alcohol drinking (Mesh-term); (2) parent* (textword), OR parenting OR parents OR parent–child relations OR family OR child rearing (Mesh-terms); (3) longitudinal OR cohort OR follow-up OR prospective OR baseline OR mixture/mixed/growth model* OR growth curve* OR generalised/generalized estimating/estimation equation* (textwords), OR GEE (title or abstract), OR longitudinal studies (MeSH-terms). If the keywords did not exist in PsycINFO or EMBASE, alternative keywords were identified in the index of the database in question.

For the selection of the studies, a list of inclusion and exclusion criteria was developed in order to detect relevant longitudinal studies. A study was included in the review if: (1) it was a prospective cohort study; (2) there were at least two assessments of alcohol use between ages 10–17 in order to quantify change in alcohol use; (3) the total follow-up period was at least one year; and (4) data on alcohol use were presented separately but not if it only presented these data for the combined use of several substances. For measurement of the PCR, we only included those studies which measured (aspects of) the PCR construct as defined in the introduction. Each possibly relevant study was checked on item-level to be sure this construct was measured. Some studies used questionnaires composed of items regarding the family in general along with items regarding the parents. These studies were included if over half of the items concerned the parents. Studies were excluded if they included only clinical populations and if alcohol use was not the outcome measurement. In addition, dissertation abstracts, reviews, comments, letters, and editorials were excluded.

The selection of the studies was performed by the three authors. First, the titles or abstracts of the identified references were screened by the first author (LV). Subsequently, for the remaining references, the full paper was retrieved and was screened regarding the selection criteria. In case of doubt, the study was discussed by the first (LV) and the second author (AFW) in order to reach consensus. If necessary, the third author (SAR) was consulted. Finally, the reference lists of all the selected publications and of relevant systematic reviews were screened for potential missing studies.

### Quality assessment

The methodological quality of the studies was assessed using a checklist derived from Hayden et al. [20]. This guideline lists criteria regarding six domains of potential biases in prognostic studies: (1) study participation; (2) study attrition; (3) predictor measurement; (4) outcome measurement; (5) confounding measurement; and (6) analysis. Sixteen relevant criteria considering each of the six potential biases were selected and adapted to the review question (Table 1). For the judgment of the validity and reliability of the measurement of alcohol use (criterion K), requirements concerning assessment of alcohol consumption were taken into account [21]. All the selected studies were independently assessed by the first two authors (LV and AFW). All criteria were rated as either “yes” (+), “partly” (±), “no” (−), or “unsure” (?) – and given 2, 1, 0, and 0 points, respectively – or as “not applicable.” In case of any disagreement, consensus was reached by discussion and, if necessary, the third author (SAR) was consulted. If ≤50% of the maximum score for a possible bias was obtained, the bias was scored as 1 (considerable risk for bias); if >50% of the maximum score was obtained, the bias was scored as 0 (low risk). For each study to obtain a total quality score, the numbers of the biases were summed, with the result ranging from a possible 0 to 6. A study was judged to be of high quality if there was a low risk for each domain of potential bias.

**Table 1:** Criteria list for methodological quality assessment

***Criteria***	
**Study participation**	
A.	The sampling frame and recruitment are adequately described, including period and place of recruitment.
B.	Inclusion and exclusion criteria are adequately described.
C.	There is adequate participation in the study by eligible individuals and sample size is sufficient. ^a^
D.	The baseline study sample (i.e., individuals entering the study) is adequately described for relevant key characteristics (at least for age and gender).
**Study attrition**	
E.	Response rate is adequate and there are no important differences between key characteristics and outcomes for participants who completed the study and those who did not (wave 1 and 2). ^a^
F.	Response rate is adequate and there are no important differences between key characteristics and outcomes for participants who completed the study and those who did not (wave 3 and follow-up). ^a^
**Predictor measurement**	
G.	A clear definition or description of the predictor measured is provided.
H.	Continuous variables are reported or appropriate (i.e., not data-dependent) cut-points are used.
I.	The predictor measurement and method are adequately valid and reliable to limit misclassification bias.
**Outcome measurement**	
J.	A clear definition or description of alcohol use is provided.
K.	Measuring and method of the outcome measurement is adequately valid and reliable to limit misclassification bias.
**Confounding measurement**	
L.	Confounders are accounted for in the study design (matching for key variables, stratification, or initial assembly of comparable groups) or in the analysis.
**Analysis**	
M.	There is sufficient presentation of data.
N.	The strategy for model building (i.e., inclusion of variables) is appropriate and is based on a conceptual framework or model.
O.	The selected model is adequate for the design of the study.
P.	There is no selective reporting of results.

^a^ An adequate participation or response rate was defined as > 80%, *or* as 60-80% and non-participation or non-response not selective.

### Best-evidence synthesis

The heterogeneity of the included studies precluded a meta-analysis to summarize the results. Therefore a best evidence synthesis was applied to determine the strength of the evidence in regard to the effects of the PCR on the change in adolescent alcohol use, as used in other reviews [22,23]. This synthesis was based on the number, the quality, and the outcome of the studies, and leading to four levels of strength for the evidence of the existence of an association between the PCR and alcohol use: (1) strong evidence: consistent findings in at least two high-quality studies; (2) moderate: consistent findings in one high-quality study and at least one low-quality study; (3) weak: findings in one high-quality study *or* consistent findings in at least three low-quality studies; and (4) inconclusive: inconsistent findings irrespective of study quality, or less than three low-quality studies available. Findings were considered to be consistent when at least 75% of the studies involved had agreed on the existence and direction of the association between the PCR and alcohol use.

## Results

### Search results

**Figure 1:**
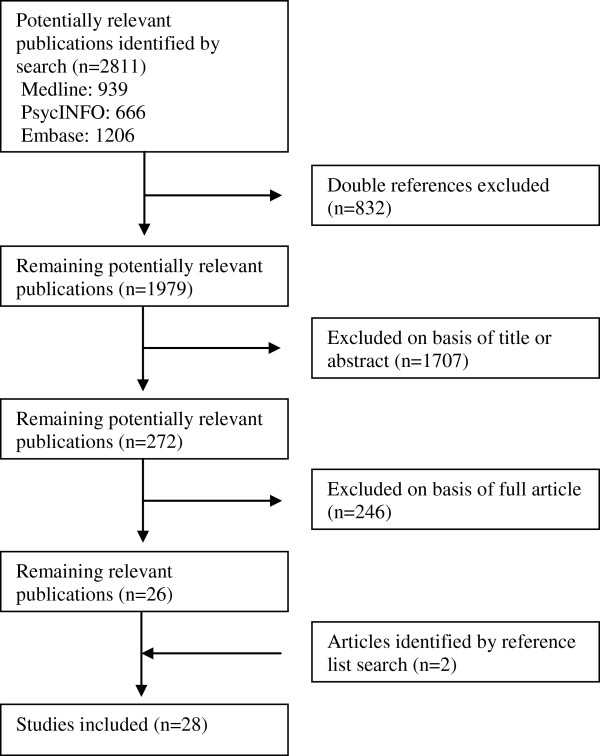
Flow chart of literature search and selection.

The search strategy resulted in 2811 references of which 28 studies were included (for detailed information about any of the steps in the screening process, see Figure 1). Fifteen of these 28 studies were not included in the review of Ryan et al. [14], 27 studies not in the review of Foxcroft and Lowe [12], and none of the studies were included in the review of Vakahali [13].

### Description of the studies

The characteristics of the studies we selected are summarized in Table 2. Twenty-one studies were undertaken in the USA, two in the Netherlands, one in Finland, one in Spain, one in New Zealand, one in Sweden, and one in Taiwan. The studies included different age groups at baseline, which varied between age 10 to over 18. The number of participants in the studies ranged from 166 to 4731. The total follow-up period ranged from 1 to 12 years and the number of waves varied from 2 to 8.

**Table 2:** Cohort studies included in review

**Author, year, country**	**Age/grade at start of study, waves, n**	**Predictor**^**a**^	**Outcome**^**a**^	**Variables accounted for in study design or analysis**	**Analysis**	**Results**
Adrados, 1995 Spain	Age 15-18+	Trust in parents.	Alcohol initiation.	Situation variables.^b^	Linear regression analysis	B= -.076, p≤.05
	2 waves in 1 year					
	n= 614					
Andrews et al., 1997 USA	Age 11-15	Quality of parent-adolescent relationship (created by summing the subscales measuring the parent’s appraisal of the adolescent and the adolescent’s appraisal of his/her parent).	Categorized as “current user” and “current nonuser”.	Gender, age, marital status	Generalized estimating equations	Father:
	6 waves in 5 years		User: currently using “at least once in a while” and a rate of use of >0 times per month over previous 6 months.			Relationship: β= -.03, ns
	n= 657					Age*relationship: β= -.64, ns
						Gender*relationship: β=-.29, ns
						Mother:
						Relationship: β= -.04, ns
			Nonuser: never used (report of never tried, along with a rate of zero times per month in last 6 months) or previous but not current users (report of quitting, along with a rate of zero times per month in last 6 months).			Age*relationship: ns
						Gender*relationship:
						β= -.27, p<.01:
						Boys: β= -.04, ns
						Girls: β= -0.31, p<.001
Aseltine & Gore, 2000 USA	9^th^-11^th^ grade^c^ 4 waves in 9 years	Parental support: degree to which the parents make the child feel loved and wanted, trust the child, and the extent to which the child enjoys being with family members.	Frequency of alcohol use during past 12 months (0= never to 7= every day).	Gender, family structure, family’s standard of living, parental education, conflict with parents, life events, peer support, peer conflict.	Hierarchical linear modeling	Frequency of use:
	n= 1208	Parent conflict: average frequency of arguments with mother and father.	Frequency of heavy alcohol use (5 or more drinks in a row) (0= never to 4= 6 or more times).			Support: B= .007, ns
						Age*support: B= .024, p<.05
						Conflict: B= .033, ns
						Age*conflict: B= -.010, ns
						Frequency of heavy use:
						Support: B= -.004, ns
						Age*support: B= .012, p<.05
						Conflict: B= .054, ns
						Age*conflict: ns
Barnes et al., 2000 USA	Age 13-16	Support: behavior toward the adolescent indicating to her or him that she or he is valued and loved.	A composite alcohol misuse index: ounces of alcohol from all beverages per day (based on frequency and quantity of consuming beer, wine, and liquor in the past year), times drunk in the past year, and frequency of having five or more drinks at a time during past year.	Gender, age, race, parental alcohol misuse.	Latent growth structural equation modeling	ns
	7 waves in 6 years					
	n= 506					
Branstetter et al., 2011 USA	10^th^ grade	Maternal support: attachment, caregiving, and affiliation	Frequency of alcohol use during past 30 days (1= never used to 8= use every day).	Gender, friend’s substance use, friendship support, friendship negative interaction, previous alcohol use	Linear regression analysis	Maternal support:
	2 waves in 1 year	Maternal negative interaction: conflict, antagonism, and criticism.				β= -.011 ns
	n=166					Maternal negative interaction:
						β= -.013 ns
Chuang et al., 2005 USA	Age 12-14	Parental closeness: attachment, involvement, child-centeredness	Categorized as: “use of alcohol” and “no use of alcohol”.	Age, gender, race/ethnicity, parents’ education, treatment condition.	Structural Equation Modeling	ns
	3 waves in 1 year					
	n= 959					
Cohen et al., 1994 USA	Cohort 1:	Positive relationship: affectional interactions.	Categorized as: “monthly user” (used alcohol in previous month) and “nonuser” (did not use alcohol in previous month).	Gender, study classification (study or control), ethnic group.	Logistic regression analysis	Cohort 1:
	5^th^ grade					5^th^ to 6^th^ grade/ 6^th^ to 7^th^ grade/ 7^th^ to 8^th^ grade, RR (95% CI)=
	4 waves in 3 years					97 (.65-1.44), ns/
	n= 618					69 (.46-1.01), ns/
	Cohort 2:					96 (.65-1.42), ns
	7^th^ grade					Cohort 2:
	3 waves in 2 years					7^th^ to 8^th^ grade/ 8^th^ to 9^th^ grade
	n= 732					(95% CI)=
						(.52-.82) p<.001/
						67 (.50-.90) p <.01
Cookston & Finlay, 2006 USA	7th -12th grade	Parent–child involvement: activities shared with parents, discussion with parents, parent–child closeness.	Mean of 3 items: how often alcohol used in past year, how often 5 drinks in a row, how often gotten drunk.	Gender, age, parent education, father status, previous alcohol use.	Structural Equation Modeling	Father’s involvement:
	waves in 1 year					β= -.04, ns
	n= 2387					Mother’s involvement:
						β= -.05, ns
Crawford & Novak, 2002 USA	10^th^ grade	Attachment: quality of child-parent relation.	Number of times consumed alcohol in their lifetime.	Gender, race, socioeconomic background, peer affiliation, participation in unstructured peer interaction, participation in structured activities, time spent with parents, parental monitoring, parental control, previous alcohol use.	Ordinary least square (OLS) and l ogistic regression analysis	Number of drinks in lifetime:
	2 waves in 2 years		Number of times for heavy drinking (5 or more drinks in a row) in past 2 weeks.			B= .00, ns
	n= 2506 (OLS analysis)/ n= 426 (logistic analysis)		Onset of alcohol use.			Times heavy drink:
			Onset of heavy drinking.			B= -.01, ns
						Onset of alcohol use:
						B= -.0072, ns
						Onset of heavy drinking:
						B= -.0872, p<.05
Danielsson et al., 2011 Sweden	7^th^ grade	Attachment: strong/secure emotional bonds to parents.	Categorized as: “yes” or “no” (reported respectively did not report heavy episodic drinking on at least one occasion).	Drinking friends, money to spend, smoking, parental provision of alcohol, bullying, truancy, time with parents, parental monitoring, previous heavy episodic drinking.	Logistic regression analysis	Boys: ns
	2 waves in 2 years		Measurement: “how often do you drink six cans of medium-strength beer, or four cans of normal beer, or four large bottles of strong cider, or a bottle of wine, or half a bottle of spirits on the same occasion?”			Girls:
	n= 1222					OR (95% CI)= 1.33 (0.85-2.09)
Donohew et al., 1999 USA	6th grade	Positive family relations: e.g., gets along with their mother/father, has fun with parents, are happy at home.	How many times alcohol used during past year (1= none to 7= 40 or more times).	Sensation seeking, attitudes toward alcohol and drugs, peer sensation seeking, perceived alcohol/marijuana use by friends, peer alcohol/ marijuana use, perceived peer influence to use alcohol/ marijuana, previous alcohol/marijuana use.	Structural equation modeling	8th to 9th grade:
	3 waves in 2 years					β= -.07, ns
	n= 428					9th to 10th grade:
						β= -.03, ns
Droomers et al., 2003 New Zealand	Age 11	Attachment: three ubscales of communication, trust and alienation.	Average amount consumed on a typical occasion categorized at each measurement as 25% highest amounts vs. 75% lowest amounts of alcohol use.	Gender, mother’s attitude towards alcohol consumption of child, friends’ attitude towards alcohol consumption, noticeable alcohol problems in family, knowledge of child about alcohol (obtained by parents), intelligence.	Generalized estimating equations	High attachment:
	5 waves in 10 years					OR= 1.00
	n= 1037					Medium attachment:
						OR= 1.42, p<.05
						Low attachment:
						OR= 1.50, p<.05
Eisenberg et al., 2008 USA	Mean age 12.8	Family connectedness: “How much do you feel your mother/father cares about you?” and “Do you feel you can talk to your mother/father about your problems?”	Categorized as: “at least monthly use” and “less frequent or nonuse.”	Race, SES, previous substance use, family meals.	Logistic regression analysis	Female:
	2 waves in 5 years		Measurement: how often used alcohol during past year (0= never to 5= daily).			OR (95% CI)= 1.31 (0.89-1.95)
	n= 806					Male:
						OR (95% CI)= 0.93 (0.65-1.34)
Ennett et al., 2001 USA	Age 12-14	Supportiveness: parent–child relationship as helping the adolescent when needed, providing encouragement and praise, and spending time together (parent report).	Categorized as: “escalators” (at least sipped at T1 and increased drinking level at T2), “initiators” (did not sip at T1 and sipped at T2) and lifetime nondrinkers (never sipped or drank any alcohol).	Gender, age, race/ethnicity, mother’s education, family structure, parent–child communication about tobacco and alcohol (rules, consequences, media), parental smoking, parental alcohol use, parental disapproval of tobacco and alcohol use, monitoring.	Logistic regression analysis	Initiation:
	2 waves in 1 year					OR = 1.78, ns
	n= 476					Escalation:
						OR = 1.71, ns
Flory et al., 2004 USA	6th grade	Family relations: how close the participant felt to their parents or guardians and the quality of these relationships.	Past month alcohol use (0= not drunk alcohol to 6= 40+).	Gender.	Latent class growth analysis (to identify subgroups) and analysis of variance (to test differences between subgroups)	F=2.67, ns
	6 waves in 10-12 years		Three subgroups determined: early onset, late onset, non-users.			
	n= 481					
Guilamo-Ramos et al., 2004 USA	7^th^ -11^th^ grade	Maternal warmth: “Most of the time my mom is warm and loving toward me.”	Heavy episodic alcohol consumption: frequency of drinking five or more drinks in a row in the past 12 months (0= never to 6= every day or almost every day).	Gender, grade.	Extension of generalized estimating equations	Three way interaction by gender*grade*warmth: p<.001:
	2 waves in 1 year					predicted means at high/medium/low level of maternal warmth for boys:
	n= 1420					1.29 REF/ 1.85, p<.05/ 2.41, p<.05
						Predicted means at high/medium/low level of maternal warmth for girls:
						1.58 REF/ 1.48, ns/ 1.38, ns
Gutman et al., 2011 USA	Age 13	Positive identification: feeling close to parents, respecting parents, wanting to be the kind of person the parent is, doing things together.	How many alcoholic drinks have you had in past 30 days (0= none to 3= one or more per week).	Gender, SES, ethnicity.	Hierarchical linear modeling	Positive identification:
	5 waves in 7 years	Negative interactions: parents criticizing ideas; putting their needs above adolescents’ needs; having hit, pushed, grabbed, or shoved adolescent.				B= -.106, p<.001
	n= 1160					Negative family interactions:
	This study: wave 1, 3, 4 and 5					B= .084, p<.001
						Time-lagged positive identification:
						B= -.107, p<.001
						Time-lagged negative family interactions:
						B= ns
Horton & Gil, 2008 USA	Mean age 11	Parent–child communication/ connectedness and attachment: sharing private thoughts and feelings with mother/father.	Frequency and level of alcohol use.	SES, family structure, previous alcohol use.	Linear regression analysis	Parent–child communication/connectedness and attachment:
	3 waves in 2.5 years	Parental derogation/rejection: being disliked by, put down by, or of little interest to one’s parents.				β= -.057, ns
	n= 451 (boys)					Parental derogation/rejection:
						β= -.038, ns
Hung et al., 2009 Taiwan	5^th^ grade	Parental support: encourages, praises, consoles, cares when sick, listens, cares about what happens at school, and helps in solving problems.	Categorized as: “first-time user” (never-user at T1 and ever-user at T2) and “never-user” (never at T1 and T2).	Gender, area, parent’s marital status/living arrangement, household income, father’s and mother’s educational level, parental alcohol use, family conflict.	Logistic regression analysis	β= -.05, p<.01
	2 waves in 1 year		Measurement: have you ever used alcohol? (1= never to 6= every day in the past month).			OR (95% CI)= .95 (.92-.99)
	n= 1183					
Kosterman et al., 2000 USA	5^th^ grade	Bonding to mother: sharing thoughts and feelings and desire to be the kind of person one’s mother is.	Alcohol initiation: the first point at which a participant reported having “ever drunk beer, wine, whiskey, gin, or other liquor.” From 5^th^ wave and follow-up question was revised to include “other than a sip or two.”	Gender, race/ethnicity, previous marijuana initiation, parents’ proactive family management, parents’ alcohol use norms, associates’ alcohol use, participants’ alcohol use norms.	Survival analysis	B= -.06, ns
	8 waves in 7,5 years					
	n= 808					
Kuntsche et al., 2009 The Netherlands	Age 14-17	Quality of parent-child relationship for both parents: e.g., “I tell my mother/father my problems and worries” and “my mother/father respects my feelings.”	Total number of alcoholic drinks in previous week during weekdays and on weekends at home and outside the home.	Gender	Structural equation modeling	Overall:
	3 waves in 2 years					β= -.07, ns
	n= 364					Low quality group:
						β=- .10, ns
						High quality group:
						β= -.22, p<.01
Latendresse et al., 2008 Finland	Age 11-12	Parental warmth: perceived home atmosphere (e.g., “warm, caring,” creative, supportive).	Drinking frequency (1= never to 9= daily).	Zygosity, sex, family structure, relational tension, shared activities, autonomy granting, parental discipline, parental monitoring, previous alcohol use.	Multiple mediation modeling	Parental warmth:
	3 waves in 6 years	Relational tension between adolescents and their parents (e.g., “unjust,” “argumentative”).				β= .00, ns
	n= 4731					Relational tension:
						β= .02, ns
Mogro-Wilson, 2008 USA	7th to 12th grade	Parental warmth: “Most of the time, my father/mother is warm and loving toward me”.	Combination of frequency of alcohol drinking and frequency of drunkenness (1= never to 7= everyday or almost every day).	Income, peer alcohol use, place of birth, language spoken at home.	Structural equation modeling	ns
	2 waves in 1 year					
	n= 1887					
Paschall et al., 2004 USA	Age 11-21	Parent-adolescent closeness: “How close do you feel to your dad/mom?” and “How much do you think he/she cares about you?”	Categorized as: “frequent” (more than once per month), “infrequent” (once per month or less) and “none.”	Gender, age, race, mother’s educational level, personal income, work intensity, mother’s involvement, alcohol use before age 14, past-year heavy drinking (frequent/infrequent).	Logistic regression analysis	Parent adolescent closeness:
	2 waves in 1 year	Parent-adolescent conflict: serious argument in past 4 weeks.	Measurement: frequency of drinking five or more drinks in a row in the past 12 months (0= never to 6= every day or almost every day).			Frequent:
	n= 4135					OR (95% CI)= .85 (.65-1.11), ns
						Infrequent:
						OR (95% CI)= .82 (.68-1.00), ns
						Parent-adolescent conflict: ns
Shelton & Van den Bree, 2010 USA	7^th^ or 8^th^ grade	Parent–child relations: e.g., “Most of the time, your mother/father is warm and loving toward you”.	A composite alcohol use index: five items about frequency and quantity.	Age, gender, maternal smoking and drinking, BMI, previous alcohol use.	Structural equation modeling	Boys:
	2 waves in 1 year					Pubertal timing:
	n= 2538					Early: B= .01, ns
						On-Time: B= .07, p<.05
						Late: B= .04, ns
						Girls:
						Pubertal timing:
						Early: B=.16, <.05
						On-Time: B=.09, <.05
						Late: B= .00, ns
Simons-Morton, 2004 USA	6th grade	Parental conflict (e.g., I have a parent who is hard for me to get along with, with whom I am often angry).	Categorized as initiators (participants who reported drinking in past 30 days at T2 and no drinking at T1) and no-drinkers (no drinking at T1 and T2).	-	Logistic regression analysis	OR (95% CI)= 1.48 (.98-2.23)
	2 waves in 1 year					
	n= 1009					
Van der Vorst et al., 2006 The Netherlands	Age 11-14	Attachment: relative degree of perceived parental security.	Combination of alcohol frequency and intensity.	Gender.	Structural equation modeling	Boys:
	3 waves in 1 year		Alcohol frequency: How often in past 4 weeks (1= every day to 6= have not been drinking).			T1-T2: β= -.007, ns
	n= 1012		Alcohol intensity:			T2-T3: β= -.076, p<.05
			How many glasses in past week, during weekdays, during weekends and inside and outside the home.			Girls:
						T1-T2: β= -.038, ns
						T2-T3: β= -.008, ns
						Total:
						T1-T2: β= -.042, ns
						T2-T3: β= -.024, ns
Wu et al., 2006 USA	Age 10-13	Maternal warmth and supportiveness: mother-child relationship, mutual trust and understanding, closeness (parent report).	A child was considered an alcohol user if child or parent reported consumption of a unit of alcohol (not just sips).	-	Analysis of variance	Maternal warmth and supportiveness:
	3 waves in 5 years	Parental discipline: various forms of punishment, including physical and verbal abuse, and withholding of affection (parent report).	Measurement: lifetime and past-year alcohol use.			Use (mean): 2.4
	n= 1119					No use (mean): 2.3
						p=.278
						Parental discipline:
						Use (mean): 0.5%
						No use (mean): 0.6%
						p=.115

Abbreviation: SES=socio-economic status.

^a^ adolescent report, unless otherwise indicated; ^b^ no further specification; ^c^ 5^th^ grade= age 10-11; ^d^ analysis sample unclear.

The majority of the studies measured the PCR as protective *or* as risk factor. In seven studies both potential roles of the PCR were measured. In two studies, the quality of the relationship was based on parent report, while in all the other studies adolescent report was used. Table 2 shows all terms and descriptions that were used for the PCR in the included studies. In the current review, the term PCR refers to all those terms.

Heterogeneity was noted as to measurement of alcohol use. Some studies provided information on the initiation of (monthly) use. Other studies provided information on the frequency and/or amount of use or on (the initiation of) heavy drinking. Few studies used other outcomes for alcohol use (e.g., a composite score of frequency of use and frequency of drunkenness). In one study, parent *or* adolescent report of alcohol use was used in the analysis; in all other studies, adolescent report was used.

A large diversity of statistical approaches was used to analyze the association. Almost half of the studies made use of “traditional” methods as analysis of variance (ANOVA) [24,25] or a form of regression analysis [26-37]. Other studies used more “sophisticated” methods as generalized estimating equations (GEE) [38-40], hierarchical linear modeling (HLM) [41,42], structural equation modeling (SEM) [43-49], latent class growth analysis (LCGA) [24], multiple mediation modeling [50] or a combination of these methods [51].

Some studies showed results that need an additional explanation. A number of the studies reported only one beta or odds ratio while the study had more than two waves [33,35,38,39,41,43,46,50]. One of these studies used only two of the three available waves without a clear reason for this [33] whereas the other study did this because neither the PCR nor alcohol use was measured at the third wave [46]. For the other studies that reported only one beta or odds ratio but the study had more than two waves, the data analytic approach provided an explanation for this. Chuang et al. [43] and Latendresse et al. [50] measured the PCR at one of the first two waves (T2 and T1, respectively) and alcohol use at the remaining two waves. In their analyses alcohol use at the last wave was predicted by the PCR, controlling for alcohol use measured at the other wave at which it was assessed in that study (T1 and T2, respectively). Andrews et al. [38] and Droomers et al. [39] did a GEE-analysis. In these GEE-analyses all waves were combined, leading to one estimate of the effect of the dependent variable on the independent variable and thus to only one beta [38] or odds ratio [39]. Kosterman et al. [35] used survival analysis which yielded only one beta based on the eight waves. Aseltine and Gore [41], and Gutman et al. [42] used HLM-analyses which were equivalent to the LGCA-analyses as done by Barnes et al. [51]. In all three studies latent growth curves were estimated resulting in one intercept and one slope for the PCR was related.

Also three other studies need an additional explanation. Wu et al. [25] used ANOVA for which F-values were not reported but p-values were given. Flory et al. [24] used LCGA with ANOVA. First a LCGA analysis was done which resulted in the identification of three subgroups: early onset, late onset, and non-users. Next ANOVA was done to test differences between the subgroups with respect to PCR. Although the focus of this systematic review was on the direct effect of the PCR on alcohol use, Barnes et al. [51] found an indirect of the PCR on alcohol misuse that operated via parental monitoring.

### Study quality

Results of the quality assessment are shown in Table 3. Nine studies (32%) met the criteria of each of the domains of potential biases and therefore were judged as high quality. The remaining 19 studies (68%) were at risk of one or more biases.

**Table 3:** Results of methodological quality assessment of included studies

**Domain**	**1**				**2**		**3**			**4**		**5**	**6**				**No. biases**
**Criterion**	**A**	**B**	**C**	**D**	**E**	**F**	**G**	**H**	**I**	**J**	**K**	**L**	**M**	**N**	**O**	**P**	
Barnes et al., 2000	+	+	?	+	+	±	±	+	+	±	+	+	±	+	+	±	0
Danielsson et al., 2011	+	+	+	+	+	NA	+	+	+	±	+	+	+	+	+	+	0
Mogro-Wilson, 2008	+	+	?	+	+	NA	+	+	±	+	+	+	±	+	+	+	0
Kuntsche et al., 2009	+	+	?	+	+	+	+	+	+	+	±	+	±	+	+	+	0
Latendresse et al., 2008	+	+	+	±	+	+	+	+	+	±	+	+	+	+	+	+	0
Shelton & Van den Bree, 2010	+	+	?	+	+	NA	+	+	+	+	+	+	+	+	+	+	0
Simons-Morton, 2004	±	+	+	+	+	NA	+	+	±	+	±	NA	±	NA	+	+	0
Van der Vorst et al., 2006	+	±	+	+	+	+	+	+	+	+	±	+	+	+	+	+	0
Wu et al., 2006	+	+	+	+	+	+	+	+	+	+	+	NA	±	NA	+	+	0
Aseltine et al., 2000	+	+	?	+	?	±	+	+	±	+	+	+	±	+	+	+	1
Chuang et al., 2005	+	+	-	+	±	±	+	+	+	+	±	+	-	+	+	+	1
Cookston & Finlay, 2006	+	+	?	±	?	NA	+	+	+	+	±	+	+	+	+	+	1
Droomers et al., 2003	+	+	+	?	+	+	+	+	+	*±*	*±*	+	±	+	+	+	1
Eisenberg et al., 2008	+	+	+	+	±	NA	+	+	+	+	±	+	-	+	+	+	1
Ennett et al., 2001	±	+	-	+	+	NA	+	+	+	±	±	+	±	+	+	+	1
Flory et al., 2004	+	+	+	+	+	-	+	+	+	+	±	+	+	+	±	+	1
Hung et al., 2009	+	+	?	+	±	NA	+	+	+	+	±	+	+	+	+	+	1
Kosterman et al., 2000	+	+	?	+	+	+	±	+	-	+	±	+	±	+	+	±	1
Paschall et al., 2004	+	+	?	+	?	NA	+	+	±	+	±	+	+	+	+	+	1
Andrews et al., 1997	±	-	NA	-	?	±	+	+	+	±	+	+	±	+	+	+	2
Branstetter et al., 2011	±	±	?	+	?	NA	+	+	+	+	±	+	-	+	+	+	2
Crawford & Novak, 2002	-	-	?	+	?	NA	+	+	±	+	±	+	+	+	?	+	2
Donohew et al., 1999	+	+	+	+	+	-	+	+	±	+	±	±	+	+	+	+	2
Guilamo-Ramos et al., 2004	+	+	?	±	?	NA	+	+	±	+	±	+	-	?	+	-	2
Gutman et al., 2011	+	±	-	+	±	±	+	+	+	+	-	+	+	+	+	±	2
Cohen et al., 1994	±	±	?	+	?	±	±	+	+	±	±	+	+	+	±	-	3
Horton & Gil, 2008	±	+	?	-	+	-	+	+	±	±	?	+	-	+	+	+	3
Adrados, 1995	+	+	?	-	-	NA	-	?	?	-	?	?	-	+	?	-	6

+ = “yes” (2), ± = “partly” (1), - = “no” (0), ? = “unsure” (0), NA = “not applicable”.

Domain: 1= study participation; 2= study attrition; 3= predictor measurement; 4= outcome measurement, 5=confounding measurement, 6= analysis

Criteria: A to P as in Table 1.

If ≤50% of the maximum score for a possible bias was obtained, the bias was scored as 1 for the domain concerned.

High quality is defined as the number of biases is 0.

There were notable limitations concerning study attrition. More than half of the studies (57%) did not have an adequate response rate, or they had important differences between participants and dropouts. Twenty-one percent of the studies were at risk of bias on the measurement of the outcome and also 21% of the studies were at risk of bias on study participation. Finally, a bias, whether on the predictor measurement, on the confounding measurement, or in the analysis, was reported in only 7% of the studies. In total, 448 items were scored twice, which resulted in disagreement on 67 items (15%), largely caused by reading errors. In all cases of disagreements, the two assessors could reach consensus during discussion.

### Association between the parent–child relationship and alcohol use

The effect of the PCR on alcohol use found in the studies included is reported in Table 2. Five studies [26,29,34,39,42], of which none was rated as high-quality, reported statistically significant negative associations between the quality of the PCR and alcohol use for the whole group. However, the findings in some of these studies [29,42] were equivocal. Crawford and Novak [29] only found a significant association for onset of heavy drinking but not for onset of alcohol use, number of drinks in lifetime, or times heavy drink. Gutman et al. [42] found significant associations in only three of four models. Those models studied the short and long-term effects of positive identification and negative interactions on alcohol use. The long-term influence of negative family interactions on alcohol use was not significant, but the remaining three associations studied were.

Seven studies [28,38,40,41,46,48,49] also found a negative association between the PCR and alcohol use, although only for specific groups. Two of these seven studies only found an effect for a certain gender [38,48], another three only for certain age groups [28,41], another two only for a certain gender with a certain age [40,49]. However, these studies did not show consistent results regarding the effect of gender or age on the association between the PCR and alcohol use. In the last study in which a subgroup analysis was performed, participants were split into a low and high-quality group for the PCR [46]. This study showed that only those who started late drinking alcohol *and* had a high-quality relationship with their parents consumed little alcohol later on. Of all these studies three were classified as high quality [46,48,49].

The remaining sixteen studies [24,25,27,30-33,35-37,43-45,47,50,51], of which six were rated as high quality [25,30,37,47,50,51], did not find a significant association in the whole group nor in a subgroup. Three of these studies [30,31,45] performed a subgroup analysis by gender or by age but did not find significant associations in one of these groups. The other thirteen studies examined the group as a whole and did not find any significant effects.

Table 4 summarizes the level of evidence for the PCR as protective (e.g., warmth, support) and as risk factor (e.g., conflict, rejection) regarding various types of alcohol behavior. We found weak evidence for the conclusion that there is an association between the PCR and frequency and/or amount of use. No systematic differences existed in the level of evidence by types of alcohol behavior. Overall, the level of evidence for the existence of an association between PCR and alcohol use seems to be stronger for PCR as protective than as risk factor.

**Table 4:** Level of evidence for the association between PCR as protective and as risk factor and types of alcohol behavior

**Parent–child relationship**	**Alcohol use**	**Consistency of findings**^**c**^**(no of findings in high quality studies)**	**Level of evidence**
Protective^a^	Initiation of (monthly) use	6 s (0), 16 ns (1)	Inconclusive
	Frequency and/or amount of use	7 s (2), 21 ns (11)	Weak
	(Initiation of) heavy drinking	4 s (0), 5 ns (2)	Inconclusive
	Other^d^	0 s (0), 5 ns (2)	Inconclusive
Total		17 (2), 47 (16)	Weak
Risk^b^	Initiation of (monthly) use	0 s (0), 2 ns (2)	Inconclusive
	Frequency and/or amount of use	1 s (0), 6 ns (1)	Inconclusive
	(Initiation of) heavy drinking	0 s (0), 2 ns (0)	Inconclusive
	Other^d^	0 s (0), 0 s (0)	Inconclusive
Total		1 s (0), 10 ns (3)	Inconclusive

Abbreviation: s=significant, ns=not significant.

^a^ Positive PCR (e.g., warmth, support).

^b^ Negative PCR (e.g., conflict, rejection).

^c^ Some studies showed more than one association and therefore total number of findings is higher than total number of studies.

^d^ Types of alcohol behavior that could not be classified in any of the other categories (e.g., a composite score of frequency of use, frequency of heavy use, and frequency of drunkenness).

### Level of evidence

According to the criteria of the best-evidence synthesis method, there is weak evidence for an effect from the PCR on change in adolescent alcohol use. The evidence was classified as weak because less than 75% of the studies agreed on the existence and direction of the relation between the PCR and alcohol use.

The effect of the PCR as protective and as risk factor on the different alcohol behaviors is shown in Table 4. The strength of the evidence for each relationship is weak to inconclusive. Overall, the level of evidence for an effect of the PCR on adolescent alcohol use is stronger for protective factors than for risk factors.

## Discussion

The aim of the current review was to summarize and determine the strength of the evidence on the effects of the PCR on change in adolescent alcohol use. Twenty-eight studies were included, nine of which were of high quality. We found weak evidence for a prospective association. The studies we included found inconsistent results: some studies found a significant negative association, while other studies did not. Previous reviews [12-14] concluded that there is a negative association between the PCR and alcohol use, whereas our estimation of the existence of an association between the PCR and alcohol use is less convincing. This difference may be explained by our decision to restrict our review to longitudinal studies with adjustment for previous alcohol use, whereas previous reviews mostly relied on cross-sectional studies or did not exclude those studies that did not adjust for previous alcohol use. This is in line with other systematic reviews reporting weaker associations between risk factors and health outcomes after controlling for confounders and adjustment for previous health problems [17]. Furthermore, we found that the effect of the PCR as protective factor was stronger than the effect of the PCR as risk factor.

The lack of at least moderate evidence for the association between the PCR and alcohol use can be explained in several ways. A first explanation is that in some studies the measuring of the PCR was poor: some studies used validated questionnaires, while other studies used *ad hoc* questions. In addition, two studies [25,32] used parent report which may be less valid than child report. Up to now, it is not clear how concordant these parent and child reports tend to be. However, it is shown that children are influenced by the parenting practices through their mental representations of it [52]. Therefore the child report may be preferred to parent report and most of the included studies did use a child report. Moreover, even we reduced the concept to a warm and supporting relationship between parent and child; this might have been insufficient to reduce heterogeneity.

Poor measurement of alcohol use may offer a second explanation for the fact that we did not find moderate or strong evidence. Some studies provided a reference period (the period over which the respondent is instructed to provide information, such as 12 months or 30 days) of little validity or even did not provide any reference period, while others used questionnaires with small sensitivity for change [21]. Furthermore, bias due to self-report of alcohol use may have added to the problem, although this is rather unlikely since self-report has been shown to be a valid method for measuring alcohol use [53,54].

A third explanation may be found in the over-adjustment of other factors. Some studies included only the PCR in a model to predict alcohol use, while other studies added factors and so examined the multivariate effects that several factors might have on alcohol use. By doing this, the association between the PCR and alcohol use may be underestimated as a result of this over adjustment. For example, to correct for parental alcohol use, may underestimate the effect of the PCR on alcohol use because it may be moderated by parental alcohol use. Higher levels of parental alcohol use might be related to a weaker association between the PCR and alcohol use by adolescents.

A fourth explanation is bias due to the attrition of participants. Some studies excluded those participants who missed one of the repeated measurements, which may have led to selection bias due to the attrition of adolescents with high levels of alcohol use. Other studies used imputation methods to reduce bias [55]. In our review we found that only three studies used an imputation method [35,45,46] thus, in the remaining studies selection bias might exist.

However, all four aforementioned explanations also apply to cross-sectional studies which show a much more consistent pattern despite this. Thus, one very likely explanation for the different findings between longitudinal and cross-sectional research is the sequence over time. A reverse causal association may exist where high levels of alcohol use cause a poor PCR. Two studies tested such a reversed association: Van der Vorst et al. [49] showed that alcohol use might have a negative effect on the PCR, while Donohew et al. [45] found no such association. Moreover, a common cause may possibly underlie the association between the PCR and alcohol use, such as having deviant peers [56-58] or overprotection on the part of parents. Deviant peers possibly influence adolescent alcohol use, but getting involved with deviant peers might also result in arguments between adolescents and their parents, which would then influence the PCR. Alcohol use might be a way to protest against parental overprotection, while overprotection might also lead to more conflicts, and so to a poor PCR.

### Strengths and limitations

Strengths of this review are the systematic evaluation of the evidence taking into account the methodological quality of individual studies and the consistency of the research findings. Another strength is the limited influence of publication bias. In our review we included studies reporting the association of the PCR with alcohol use although this was not the main research question. With this inclusion also non-significant results were taken into account. Despite of the comprehensive search strategy, a limitation is that we might have missed some studies due to the restriction imposed by using only those studies published in English. However, most reviews use a language restriction and no evidence was found that such a restriction leads to a bias [59]. Furthermore, due to restriction of the search to electronic databases and to year of publication, we might have missed some studies. But it is unlikely that unpublished studies, non-journal studies, or studies published before 1985 would influence our conclusion regarding the level of evidence. Besides that, the likelihood that we have missed some studies is small because we screened the reference lists of the selected studies and of relevant systematic reviews. Another limitation of our study may be the use of a cut-off point for the identification of high-quality studies which we used to determine the strength of the evidence. A low risk for a bias was supposed if >50% of the maximum score for that bias was obtained; this resulted in nine high-quality studies. With a cut-off point of ≥50% sixteen studies would have been rated as high quality. Nevertheless, due to the lack of consistent results (less than 75% of the studies found significant associations), the conclusion regarding the level of evidence would end up being the same.

### Implications

We found weak evidence for a causal association between the PCR and alcohol use. The causality of this association and, if causal, its direction is far from clear. It might also be for example that the PCR is only causally related to certain types of alcohol behaviors. Our study found stronger evidence for an association of the PCR with frequency and/or amount of use than with other types of alcohol use. However, research that studied the association for several types of alcohol behaviors, so the comparison of effects is more reliable, is lacking. Additional research may clarify this. Furthermore, additional research should focus in particular on the role of overprotection by parents regarding the relation between the PCR and alcohol use as discussed above. Considering the gap in the literature, this research should also concern the extent of parental influence across several stages of alcohol use, and the additional effects of peer behavior. Any future research should further be designed in such a way that it also enables the assessment of potential reversed causality. Furthermore, for progress in this area more standardization in the measurement of both PCR and alcohol use, the follow-up periods, and analytic procedures is needed, considering the significant heterogeneity present in our review.

## Conclusion

Regarding reduction of alcohol consumption, the development of prevention and intervention programs by policymakers aimed at improving the PCR does not seem to be useful, given the unclear causality. However, assessment of the PCR as protective factor may be a means to identify groups of adolescents at risk for use of alcohol, also independent from the still unclear causal mechanims. Considering the size of the problem of adolescent alcohol use, this topic deserves attention given its impact on adolescent health.

## Competing interests

The authors declare that they have no competing interests.

## Authors’ contributions

LV developed the protocol for the study, conducted the literature search, screened studies for inclusion in the review, assessed the quality of the studies, extracted data, and wrote the final manuscript. AFW was involved in the development of the protocol of the study, screened studies for inclusion in the review, and assessed the quality of the studies. SAR was involved in the development of the protocol of the study and contributed to the screening and quality assessment of the studies. All authors contributed to the interpretation of the data, writing of the manuscript and have read and approved the final manuscript.

## Pre-publication history

The pre-publication history for this paper can be accessed here:

http://www.biomedcentral.com/1471-2458/12/886/prepub
